# Interstitial Lung Disease and Pulmonary Arterial Hypertension Screening Practices in Systemic Sclerosis: Survey Insights of Rheumatologists in the Middle East and North Africa Region

**DOI:** 10.31138/mjr.211224.alf

**Published:** 2025-08-18

**Authors:** Rajaie Namas, Sarah Al Qassimi, Jawahir Alameri, Samar Al Emadi, Nelly Ziade, Ahlam Almarzooqi, Farida Al Balushi, Mohammed A. Omair, Taha Qardaghi, Yasameen Abbas Humadi, Mohamed Alawlaqi, Hanan Al Rayes, Saadeya Naji, Fatima Haji, Fajer Altamimi, Mansour Alazmi, Hani Shatnawi, Waleed Hafiz, Deena Ahmed, Mariam Almansoori, Jamal Al Saleh, Hazem Rifaai, Mahdi Abusalameh, Zaki Abou Zahr, Hiba Khogali, Sehriban Diab, Maha Anbar, Adeeba Al Herz, Amal Elganzoury, Shaima Ewila, Basant Elnady, Wafa Madanat, Imad Uthman, Asia Mubashir, Mohamed Elarabi, Suzan Attar

**Affiliations:** 1 Division of Rheumatology, Department of Internal Medicine, Medical Subspecialties Institute, Cleveland Clinic Abu Dhabi, Abu Dhabi, United Arab Emirates;; 2 Rheumatology Division, Medicine Department, Hamad Medical Corporation, Doha, Qatar;; 3 Rheumatology Department, Hotel-Dieu de France Hospital, Beirut, Lebanon;; 4 Department of Rheumatology, Al Qassimi Hospital, Emirates Health Services, Sharjah, United Arab Emirates;; 5 Department of Rheumatology, Royal Hospital, Muscat, Oman;; 6 Rheumatology Unit, Department of Medicine, King Saud University, Riyadh, Saudi Arabia;; 7 Department of Rheumatology, Sulaimanyiah Rheumatology and Medical Rehabilitation Centre, Iraq;; 8 Department of Rheumatology and Medical Rehabilitation, Alnahrain University College of Medicine, Baghdad, Iraq;; 9 Division of Rheumatology, Department of Internal Medicine, Prince Sultan Military Medical City, Riyadh, Saudi Arabia;; 10 Department of Rheumatology, Salmaniya Medical Hospital, Manama, Bahrain;; 11 Department of Internal Medicine and Rheumatology, AlSalam Specialist Hospital, Manama, Bahrain;; 12 Department of Rheumatology, Bahrain Royal Medical Services, Manama, Bahrain;; 13 Department of Rheumatology, Prince Mohammed Medical City, Aljouf, Saudi Arabia;; 14 Department of Rheumatology, Princess Basma Teaching Hospital, Irbid, Jordan;; 15 Department of Medicine, College of Medicine, Umm Al-Qura University, Makkah, Saudi Arabia;; 16 Department of Rheumatology, Sheikh Shakhbout Medical City, Abu Dhabi, United Arab Emirates;; 17 Department of Rheumatology, Dubai Hospital, Dubai Health, Dubai, United Arab Emirates;; 18 Department of Rheumatology, MediClinic Hospital, Abu Dhabi, United Arab Emirates;; 19 Department of Rheumatology, Healthpoint Hospital, Abu Dhabi, United Arab Emirates;; 20 Department of Rheumatology, Tawam Hospital, Abu Dhabi, United Arab Emirates;; 21 Department of Rheumatology, Kuwait Hospital, Kuwait City, Kuwait;; 22 Department of Rheumatology, Al-Amiri Hospital, Kuwait City, Kuwait;; 23 Department of Physical Medicine, Rheumatology and Rehabilitation, Ain Shams University, Cairo, Egypt;; 24;Rheumatology Committee, Universal Health Insurance, Port Said, Egypt;; 25 Rheumatology Department, Al Hada Armed Forces Hospital, Taif, Saudi Arabia;; 26 Department of Rheumatology, Khalidi Hospital, Amman, Jordan;; 27 Division of Rheumatology, Department of Internal Medicine, American University of Beirut, Beirut, Lebanon;; 28 Department of Medicine, King Abdulaziz University, Jeddah, Saudi Arabia

**Keywords:** systemic sclerosis, pulmonary arterial hypertension, interstitial lung disease, screening, surveys and questionnaires

## Abstract

**Background:**

Interstitial lung disease (ILD) and pulmonary arterial hypertension (PAH) are significant causes of morbidity and mortality in systemic sclerosis (SSc). Our objective was to determine screening practices for ILD and PAH in patients with SSc among rheumatologists in the Middle East and North Africa (MENA) region.

**Methods:**

An online questionnaire was distributed to rheumatologists across the MENA region. Participants were asked to estimate the proportion of SSc patients in their practice, indicate whether they screen for ILD and PAH, and specify how often they utilise screening modalities, including chest X-ray (CXR), high-resolution CT (HRCT), pulmonary function tests (PFTs), echocardiograms (ECHO), and the use of the DETECT algorithm. Data were analysed using descriptive statistics, with p-values ≤0.05 regarded as statistically significant.

**Results:**

394 respondents completed the questionnaire from 17 MENA countries. 389 (98.73%) reported screening for ILD, and 369 (93.65%) screened for PAH. 270 respondents performed screening at the time of SSc diagnosis (68.53%). Screening approaches for ILD included CXR (211, 53.55%), HRCT (321, 81.47%), and PFTs (299, 75.89%), while PAH screening included ECHO (346, 87.82%), and the DETECT algorithm (26.14%). Statistical differences were observed in the average number of SSc patients seen per year (p=0.0008), gender of respondents (p=0.02), current age (p=0.02), background training (p<0.00001), use of ECHO as PAH screening (p=0.01), and the DETECT algorithm (p=0.000017) between SSc experts and non-SSc experts.

**Conclusions:**

Our findings reflect real-world screening practices in SSc patients, emphasising discrepancies which could stem from variations in clinical training, resource availability, or institutional protocols across healthcare settings.

## INTRODUCTION

Screening is the systematic application of tests to identify individuals at risk of developing specific disorders, enabling earlier interventions and improving patient outcomes.^1^ For patients with systemic sclerosis (SSc), a rare and complex autoimmune disease marked by autoimmunity, vasculopathy, and progressive fibrosis, early and proactive screening is crucial. Given the condition’s severe multi-organ involvement, timely detection is especially important for identifying complications affecting the lungs and heart.^2^ Among the various complications associated with SSc, interstitial lung disease (ILD) and pulmonary arterial hypertension (PAH) are recognised as leading causes of morbidity and mortality. Together, they account for over 60% of deaths in patients with SSc.^3,4^ Given the serious prognosis of these complications, early detection is critical for improving survival rates and quality of life.

Despite the availability of global data on the prevalence and management of SSc-associated ILD (SSc-ILD) and PAH, there remains a significant gap in understanding how rheumatologists in the Middle East and North Africa (MENA) region approach screening for these conditions. To date, no comprehensive assessment has been conducted to evaluate the screening practices for ILD and PAH in SSc patients within this region. This is particularly important given that the healthcare practices and resource availability vary significantly across different regions, and the applicability of global guidelines may be limited in regions with unique healthcare challenges.

Given the region’s distinct healthcare challenges, including resource constraints and varying access to advanced diagnostics, assessing existing screening practices is essential. This study aims to bridge the knowledge gap by conducting the first detailed analysis of screening practices for ILD and PAH in SSc patients among rheumatologists in the MENA region. By highlighting trends, identifying disparities, and addressing gaps in current practices, it seeks to establish a foundation for future initiatives to improve and standardise SSc management in the region.

## METHODS

An online questionnaire was created using Google Forms following a literature review of published studies with similar survey designs (**Appendix**). It was then reviewed by an SSc expert and two additional rheumatologists at the corresponding author’s hospital. Between December 7, 2023, and June 14, 2024, the questionnaire was distributed to rheumatologists across the MENA region via regional rheumatology societies, registered email lists, and WhatsApp groups. The questionnaire was distributed through various rheumatology societies, including the Emirates Rheumatology Society, Qatar Rheumatology Association, Bahrain Rheumatology Society, Oman Society of Rheumatology, Saudi Society of Rheumatology, Jordan Society of Rheumatology, Syrian Association for Rheumatology, Lebanese Society of Rheumatology, Kuwait Association for Rheumatology, and the Egyptian Society of Rheumatic Diseases. Demographic information was collected from each respondent, who were also asked to self-identify as either “experts in SSc” or “non-experts in SSc.” Experts were defined as rheumatologists with formal training in SSc, publications in the field of rheumatology, or a dedicated clinic for SSc. Respondents were asked to estimate the proportion of SSc patients in their practice and whether they screen for ILD and PAH. They were also asked when they conduct screening for these conditions and how often they use specific screening methods in SSc patients, including chest X-ray (CXR), high-resolution CT (HRCT), pulmonary function tests (PFTs), echocardiograms (ECHO), and the DETECT algorithm. The DETECT algorithm is a two-step, internally validated process for screening PAH in patients with SSc.^5^ In Step 1, the patient is evaluated for non-echocardiographic variables and receives a risk score based on these variables. These include the FVC and DLCO percentage predicted, current and past telangiectasias, serum anticentromere antibodies, serum N-terminal probrain natriuretic peptide, serum urate, and right axis deviation (electrocardiogram). The total score of Step 1 determines whether the patient should be referred to echocardiography and thus undergo Step 2. In Step 2, two echocardiographic variables (right atrium area and tricuspid regurgitant jet velocity) are evaluated and combined with the previous risk score from Step 1. Step 1 can also directly be combined with Step 2. This overall risk score then recommends an RHC to confirm suspected PAH (5). Additionally, a subgroup was identified as SSc experts were also analysed and compared to non-SSc experts. Participation in the questionnaire was voluntary, with consent implied upon proceeding with the answers. No identifying information was collected, and respondents could terminate the questionnaire at any time. The project received approval from the Cleveland Clinic Abu Dhabi Research Ethics Committee (REC) under the study number A-2020-034 that was initially approved in 2020 with yearly extension that was last approved on May 6^th^, 2024.

### Statistical Analysis

Data were analysed using descriptive statistics, and comparisons were conducted with the Chi-square test and non-parametric statistical methods where appropriate using GraphPad QuickCalcs website, https://www.graphpad.com/. Differences with p-values of 0.05 or less were considered statistically significant.

## RESULTS

**Figure 1.**
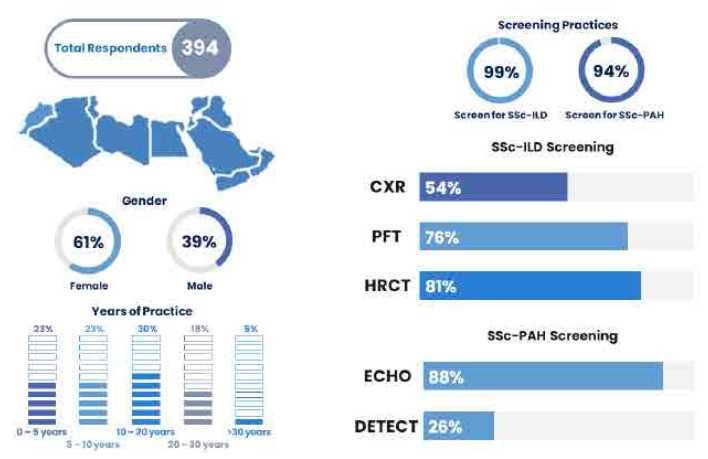
Survey respondents' demographic data and screening practices for SSc-ILD and SSc-PAH.

A total of 394 respondents from 17 countries across the MENA region completed all or part of the questionnaire (**Figure 1**). The majority of respondents were general rheumatologists, accounting for 342 (86.80%), followed by 29 rheumatology subspecialty experts (7.36%), 19 experts in SSc (4.82%), and 3 rheumatology fellows-in-training (0.76%). A higher proportion of female respondents was observed, with 241 (61.17%) compared to 152 (38.58%) male respondents. The majority of respondents were between the ages of 30 and 40 years (162, 41.12%). Most respondents (n = 258) completed their rheumatology training in the MENA region (65.48%), followed by 63 in Europe (15.99%), 38 in North America (9.64%), and 30 in Asia (7.61%). Approximately a quarter of respondents (n = 120) have been practicing for 10 to 20 years (30.46%) (**Table 1**). On average, respondents reported seeing 14 ± 16 (mean ± SD) patients with SSc annually. The majority of respondents (389, 98.73%) indicated that they screen for SSc-ILD, and 369 (93.65%) screen for PAH (**Table 2**). Over half of the respondents (270, 68.53%) conduct screening at the time of SSc diagnosis. For SSc-ILD, screening methods included CXR (211, 53.55%), HRCT (321, 81.47%), and PFTs (299, 75.89%). For PAH, ECHO was used by 346 respondents (87.82%), while only 103 respondents (26.14%) employed the DETECT algorithm for PAH screening.

**Table 1.** Demographic data of all survey respondents.

**Demographic variables**	**(n = 394)**
Current Position, n (%)	
*Rheumatology fellow-in-training*	3 (0.76)
*General rheumatologist*	342 (86.80)
*Systemic sclerosis expert*	19 (4.82)
*Expert in other rheumatological diseases*	29 (7.36)
*Unanswered*	1 (0.25)
Gender, n (%)	
*Female*	241 (61.17)
*Male*	152 (38.58)
*Unknown*	1 (0.25)
Age, n (%)	
*20 – 30 years*	21 (5.33)
*30 – 40 years*	162 (41.12)
*40 – 50 years*	126 (31.98)
*>50 years*	84 (21.32)
*Unanswered*	1 (0.25)
Background rheumatology training, n (%)	
*Middle East and North Africa*	258 (65.48)
*Asia*	30 (7.61)
*Europe*	63 (15.99)
*North America*	38 (9.64)
*Australia*	1 (0.25)
*Unanswered*	4 (1.02)
Years of practice, n (%)	
*0 – 5 years*	90 (22.84)
*5 – 10 years*	89 (22.59)
*10 – 20 years*	120 (30.46)
*20 – 30 years*	72 (18.27)
*>30 years*	18 (4.57)
*Unanswered*	5 (1.27)
Country of practice, n (%)	
*Algeria*	16 (4.06)
*Bahrain*	11 (2.79)
*Egypt*	83 (21.07)
*Iraq*	31 (7.87)
*Jordan*	17 (4.31)
*Kuwait*	8 (2.03)
*Lebanon*	4 (1.02)
*Libya*	1 (0.25)
*Morocco*	1 (0.25)
*Oman*	12 (4.31)
*Palestine*	16 (4.06)
*Qatar*	22 (5.58)
*Saudi Arabia*	52 (13.20)
*Syria*	12 (3.30)
*Tunisia*	1 (0.25)
*United Arab Emirates*	83 (21.07)
*United Kingdom*	1 (0.25)
*Yemen*	1 (0.25)
*Unanswered*	16 (4.06)

**Table 2.** SSc screening practices among all survey respondents.

**SSc screening practices, n (%)**	**(n = 394)**
Estimated number of SSc patients seen per year, mean ± SD	14 ± 16
Do you screen SSc patients for ILD?	
*Yes*	389 (98.73)
*No*	4 (1.02)
Do you screen SSc patients for PAH?	
Yes	369 (93.65)
*No*	23 (5.84)
When do you screen SSc patients for ILD/PAH?	
*Annually*	7 (1.78)
*At the time of diagnosis*	270 (68.53)
*Depends on serological biomarkers or autoantibody profile regardless of time of diagnosis*	39 (9.90)
*Only at the time patients develop symptoms such as fatigue, dyspnea, cough, etc*	74 (18.78)
*Unanswered*	4 (1.02)
Do you screen SSc-ILD with CXR?	
*Yes*	211 (53.55)
*No*	179 (45.43)
Do you screen SSc-ILD with HRCT?	
*Yes*	321 (81.47)
*No*	70 (17.77)
Do you screen SSc-ILD with PFTs?	
*Yes*	299 (75.89)
*No*	93 (23.60)
Do you screen PAH with ECHO?	
*Yes*	346 (87.82)
*No*	46 (11.68)
Do you screen PAH with the DETECT algorithm?	
*Yes*	103 (26.14)
*No*	286 (72.59)

A comparison of demographic profiles between SSc experts and non-experts revealed significant differences in several areas: the average number of SSc patients seen per year (p = 0.0008), gender (p = 0.024697), current age (p = 0.02), and background training (p < 0.00001). There was also a notable difference in years of practice, with nearly half of the SSc experts (42.11%) having at least 10 to 20 years of experience, compared to a lower proportion of non-SSc experts (**Table 3**). Regarding screening practices, the timing of screening for ILD and PAH, as well as screening for ILD using CXR, HRCT, and PFTs, did not show significant differences. However, significant differences were observed in PAH screening practices, with ECHO showing a p-value of 0.01 and the DETECT algorithm a p-value of 0.000017(**Table 4**). A summary of the findings is illustrated in **Figure 1**.

**Table 3.** Demographic profiles for rheumatologists who are experts in SSc compared to those who are non-experts in SSc.

**Variables**	**Systemic sclerosis expert**	**Non-systemic sclerosis expert**	**p-value (<0.05)**
Number of respondents, n (%)	19 (4.82)	375 (95.18)	
Average number of SSc patients/year (Mean±SD)	25.47±33.29	13.07±14.26	**0.0008**
Gender, n (%)			
*Female*	7 (36.84)	234 (62.40)	**0.024697**
*Male*	12 (63.16)	140 (37.33)	
*Unanswered*	0 (0)	1 (0.27)	
Age, n (%)			
*20 – 30 years*	0 (0)	21 (5.60)	**0.021023**
*30 – 40 years*	5 (26.32)	157 (41.87)	
*40 – 50 years*	12 (63.16)	114 (30.40)	
*>50 years*	2 (10.53)	82 (21.87)	
*Unanswered*	0 (0)	1 (0.27)	
Background training, n (%)			
*Middle East and North Africa*	5 (26.32)	253 (67.47)	**< 0.00001**
*Asia*	0 (0)	30 (8)	
*Europe*	1 (5.26)	62 (16.53)	
*North America*	12 (63.16)	26 (6.93)	
*Australia*	0 (0)	1 (0.27)	
*Unanswered*	1 (5.26)	3 (0.80)	
Years of practice, n (%)			
*0 – 5 years*	1 (5.26)	89 (23.73)	0.439869
*5 – 10 years*	5 (26.32)	84 (22.40)	
*10 – 20 years*	8 (42.11)	112 (29.87)	
*20 – 30 years*	4 (21.05)	68 (18.13)	
*>30 years*	5 (26.32)	18 (4.80)	
*Unanswered*	1 (5.26)	4 (1.07)	

**Table 4.** A comparison of variations in rheumatologists who are experts in SSc and rheumatologists who are non-experts in SSc in terms of SSc screening practices.

**SSc screening practices, n (%)**	**Systemic sclerosis expert**	**Non-systemic sclerosis expert**	**p-value (<0.05)**
Number of respondents, n (%)	19 (4.82)	375 (95.18)	
When do you screen SSc patients for ILD/PAH?			
*Annually*	0 (0)	7 (1.87)	
*At the time of diagnosis*	19 (100)	251 (66.93)	
*Depends on serological biomarkers or autoantibody profile regardless of time of diagnosis*	0 (0)	39 (10.40)	
*Only at the time patients develop symptoms such as fatigue, dyspnea, cough, etc*	0 (0)	74 (19.73)	
*Unanswered*	0 (0)	4 (1.07)	
Do you screen SSc patients for ILD?			
*Yes*	10 (100)	370 (98.67)	
*No*	0 (0)	5 (1.33)	
Do you screen SSc patients for PAH?			
Yes	19 (100)	350 (93.33)	
*No*	0 (0)	25 (6.67)	
Do you screen SSc-ILD with CXR?			
*Yes*	5 (26.32)	206 (54.93)	0.149924
*No*	14 (73.68)	169 (45.07)	
Do you screen SSc-ILD with HRCT?			
*Yes*	19 (100)	302 (80.53)	
*No*	0 (0)	73 (19.47)	
Do you screen SSc-ILD with PFTs?			
*Yes*	16 (84.21)	283 (75.47)	0.384709
*No*	3 (15.79)	92 (24.53)	
Do you screen PAH with ECHO?			
*Yes*	19 (100)	327 (87.20)	**0.013548**
*No*	0 (0)	48 (12.80)	
Do you screen PAH with the DETECT algorithm?			
*Yes*	13 (68.42)	90 (24)	**0.000017**
*No*	6 (31.58)	285 (76)	

## DISCUSSION

A collaborative study in the UAE has offered insights into the prevalence of SSc and its associated visceral involvement within the region, revealing a prevalence rate of 1.66 per 100,000 in the general population and 7.78 per 100,000 among patients in the United Arab Emirates.^4^ Within this cohort, 53.30% of patients were diagnosed with SSc-ILD and 17.36% with PAH, highlighting the significant burden of these complications. Additionally, in a study that analysed the mortality of SSc in a prospective multinational EUSTAR cohort, the results show a high prevalence of SSc-related causes of death with a high prevalence of ILD, PAH, and myocardial causes, respectively (6). Beyond these findings, there is limited information on how frequently or systematically rheumatologists in the MENA region screen for these life-threatening complications.

The survey results provide valuable insights into SSc screening practices within the MENA region, marking the first study to specifically gather data from rheumatologists in the Middle East and North Africa on their approaches to screening for SSc-ILD and PAH. This study highlights current practices and variations, offering a foundational understanding of how these conditions are monitored in this geographic area. By identifying trends and gaps in screening practices, the study sets the stage for future improvements and standardisation in the management of SSc across the region. Overall, the survey data suggests a degree of heterogeneity in screening for both SSc-ILD and PAH. For example, while 68.53% of respondents screen for ILD and PAH at the time of SSc diagnosis, the rest perform screening based on different criteria: 18.78% only when patients show symptoms, 9.90% based on serological biomarkers or autoantibody profiles, and 1.78% on an annual basis. These practices align with findings from a global survey by Bernstein et al.^7^ which reported that 51% of general rheumatologists and 66% of SSc experts routinely ordered HRCTs for all newly diagnosed SSc patients. The study also noted that HRCT screening was often prompted by the development of ILD or PAH symptoms (100% of SSc experts versus 80% of general rheumatologists) and the presence of certain autoantibodies, such as anti-Scl-70 (38% versus 32%) and anti-RNA polymerase 3 (30% versus 14%).^7^

This study not only sheds light on the current state of screening practices in the MENA region but also emphasises the need for harmonised guidelines tailored to the specific healthcare landscape in this area. Disparities in ILD screening were evident in our questionnaire, particularly in the varying use of CXR, HRCT, and PFTs among respondents. The most recent expert consensus recommends using chest auscultation, comprehensive PFTs, spirometry with DLCO, HRCT, and autoantibody testing for screening SSc patients for ILD.^8^ Although CXR is not included in this consensus, 53.55% of respondents reported using it for ILD screening, with no significant differences between SSc experts and non-SSc experts. This may be attributed to the wider availability of plain chest X-rays compared to HRCT or PFTs, which require specialised facilities and trained physicians. As for HRCT and PFTs, the majority of respondents reported utilising HRCT (81.47%) and PFTs (75.89%) in their practice. Although HRCT is the gold standard for ILD diagnosis, an international survey revealed that only 65% of physicians routinely use it for screening at SSc diagnosis. Non-screeners cited various reasons, including reliance on clinical suspicion, concerns about radiation exposure, the need for stronger scientific evidence, as well as cost and administrative barriers.^9^ It is important to note that while PFTs are commonly used, their high false-negative rate and low sensitivity for detecting SSc-related ILD mean they should be used in conjunction with HRCT for effective screening.^10^ In a study evaluating the performance of PFTs compared to HRCT in the detection of SSc-ILD, 53% of 75 patients with normal FVC values showed significant ILD on HRCT, therefore when FVC alone was used for screening and early detection of ILD, there was a false-negative rate of up to 62.5%.^11^ These results are in keeping with previous studies that confirm a discordance of SSc-ILD between PFTs and HRCT.^12,13^

These disparities likely reflect both resource limitations and differences in clinical training across the region. In many parts of the MENA region, advanced diagnostic tools such as HRCT and PFTs may not be readily available, leading clinicians to rely on CXR, which is more accessible but less effective. This points to the need for broader access to specialised diagnostic tools, particularly in resource-constrained environments. Regional initiatives that improve healthcare infrastructure and provide ongoing education to healthcare professionals could address these gaps.

Our findings highlighted significant differences in PAH screening practices between SSc experts and non-SSc experts. SSc experts were more likely to use ECHO and the DETECT algorithm for screening. ECHO is currently used to diagnose PAH in SSc patients, as studies have shown that it can detect PAH at earlier and milder stages, leading to better outcomes.^14^ Additionally, a cohort analysis comparing PAH screening guidelines from the European Society of Cardiology/European Respiratory Society found that combining the DETECT algorithm with ECHO yielded the highest detection rates for PAH compared to using either method alone.^15^ This underscores the importance of emphasising the combined use of ECHO and the DETECT algorithm as a more effective screening strategy for PAH in SSc within our region.

As anticipated, there was a statistically significant difference in the number of SSc patients seen annually between SSc experts and non-experts, with SSc experts managing a higher volume of patients. Additionally, differences were observed in the age, gender, and background training of rheumatologist respondents. Gender disparities also emerged, reflecting variations in professional experience and expertise. These factors collectively highlight the influence of experience and training on the volume and nature of patient care provided in the field of systemic sclerosis.

Our findings reflect current real-world practices for screening ILD and PAH in SSc patients and reveal significant variations in these practices across the region. These differences highlight the need for standardisation to improve and unify screening methods. Standardising screening protocols could enhance early detection and management of ILD and PAH, leading to better patient outcomes. Uniform guidelines would ensure that all patients receive consistent care, reducing discrepancies between different healthcare providers and settings. This alignment can improve the reliability of early diagnosis and treatment, ultimately benefiting patient health. Additionally, addressing these variations will help identify and implement best practices, establish regional screening protocols, and ensure that patients have access to the most effective and up-to-date screening techniques. This approach aims to provide equitable, high-quality care for SSc patients throughout the region.

Moreover, this study serves as a baseline for future research and quality improvement initiatives aimed at enhancing the management of SSc in the MENA region. Establishing a larger regional registry for SSc patients, for instance, could provide valuable longitudinal data that would further refine screening practices and treatment outcomes. Additionally, education campaigns tailored to general rheumatologists in the region could help improve adherence to best practices for screening and early detection. These steps would be crucial in closing the gaps between SSc experts and non-experts and ensuring that all patients, regardless of location, benefit from the most advanced and evidence-based care. Several limitations should be considered when interpreting the findings of this study. First, there is great heterogeneity of the sample and may not fully represent all rheumatologists in the MENA region, as participation was voluntary, and responses were gathered via an online questionnaire, which may have excluded certain populations. Additionally, the self-reporting nature of the questionnaire introduces the potential for response bias, as respondents may have overestimated their screening practices. The study also did not examine the impact of screening practices on clinical outcomes, which would require longitudinal data to assess the effectiveness of various screening protocols. Finally, while the survey provided valuable insights, it may not capture all factors influencing screening decisions, such as institutional policies or local healthcare infrastructure. Further studies with broader representation and longitudinal follow-up are needed to validate and expand on these findings.

In conclusion, this pioneering study provides a foundational understanding of current screening practices for ILD and PAH among rheumatologists in the MENA region, highlighting both the strengths and gaps in care. By addressing the disparities identified in this study, healthcare systems in the region can work toward more standardised and effective screening protocols, ultimately improving the early detection and management of SSc-related complications. Through regional collaboration, infrastructure improvement, and adherence to international best practices, the quality of care for SSc patients across the MENA region can be significantly enhanced.

## AUTHOR CONTRIBUTIONS

All authors take full responsibility for the integrity and accuracy of all aspects of the work. Specific contributions are as follows:

Rajaie Namas: Conception, analysis, literature review, writing, submission, supervision, revision.

Sarah Al Qassimi: Conception, analysis, literature review, writing, submission, supervision, revision.

Jawahir Alameri: Literature review, writing.

Samar Al Emadi: Writing, supervision.

Nelly Ziade: Writing, supervision.

Ahlam Almarzooqi: Conception, literature review, writing, submission, supervision, revision.

Farida Al Balushi: Literature review, supervision.

Mohammed A. Omair: Literature review, supervision, writing.

Taha Qardaghi: Literature review, supervision.

Yasameen Abbas Humadi: Literature review, writing.

Mohamed Alawlaqi: Literature review.

Hanan Al Rayes: Literature review, supervision.

Saadeya Naji: Literature review, supervision.

Fatima Haji: Literature review, supervision.

Fajer Altamimi: Literature review, supervision.

Mansour Alazmi: Literature review.

Hani Shatnawi: Literature review, supervision.

Waleed Hafiz: Literature review.

Deena Ahmed: Literature review.

Mariam Almansoori: Literature review.

Jamal Al Saleh: Literature review, supervision.

Hazem Rifaai: Literature review.

Mahdi Abusalameh: Literature review.

Zaki Abou Zahr: Literature review.

Hiba Khogali: Literature review.

Sehriban Diab: Literature review.

Maha Anbar: Literature review.

Adeeba Al Herz: Literature review, writing.

Amal Elganzoury: Literature review, supervision.

Shaima Ewila: Literature review.

Basant Elnady: Literature review, supervision.

Wafa Madanat: Literature review, supervision.

Imad Uthman: Literature review, supervision.

Asia Mubashir: Literature review.

Mohamed Elarabi: Literature review.

Suzan Attar: Literature review, supervision, writing.

## CONFLICTS OF INTEREST

All authors declare no conflicts of interest.

## FUNDING

No funding required for this study.

## ETHICS APPROVAL

This study received ethics approval from the Cleveland Clinic Abu Dhabi Research Ethics Committee (REC) under the study number A-2020-034.

## NOTE

This study was presented as a poster presentation at the 14^th^ Advanced Academic Rheumatology Review Course.

## APPENDIX

### Appendix

Systemic Sclerosis Screening for Organ Involvement Thank you for agreeing to answer the following survey surrounding screening for interstitial lung disease and pulmonary arterial hypertension in patients with systemic sclerosis in the Middle East and North African region with an aim to understand the screening practices in our region.

The survey should take no longer than 3 minutes to complete. Kindly answer all questions to the best of your ability. Your responses are anonymous as no identifying information is being collected. By submitting your answers to the survey, you are consenting to having your responses collected. We thank you for your participation.

**
Physician Demographic Data
**

I am a

○ General rheumatologist
○ Systemic sclerosis (SSc) expert
○ Expert in other rheumatological diseases
○ Others

Gender

○ Female
○ Male

Age

○ 20–30 years old
○ 30–40 years old
○ 40–50 years old
○ >50 years old

Background rheumatology training

Background rheumatology training

○ Middle East and North Africa
○ North America
○ Europe
○ Asia
○ Other

Country of current practice

______________________

Years of practice

○ 0–5 years
○ 5–10 years
○ 10–20 years
○ 20–30 years
○ >30 years

Average number of SSc patients seen in clinic per year

______________________

**
Screening Methods
**

Do you screen SSc patients for ILD?

○ Yes
○ No

Do you screen SSc patients for PAH?

○ Yes
○ No

When do you screening SSc patients for ILD/PAH?

○ At time of diagnosis
○ Only at the time patients develop symptoms such as fatigue, dyspnea, cough etc
○ Depends on serological biomarkers or autoantibody profile regardless of time of diagnosis
○ Other

Do you screen for ILD in SSc patients with CXR?

○ Yes
○ No

Do you screen for ILD in SSc patients with HRCT?

○ Yes
○ No

Do you screen for ILD in SSc patients with PFTs?

○ Yes
○ No

Do you screen for PAH in SSc patients in ECHO?

○ Yes
○ No

Do you screen for PAH in SSc patients using the DETECT algorithm?

○ Yes
○ No
